# Alzheimer’s Disease Polygenic Risk Score Is Not Associated With Cognitive Decline Among Older Adults With Type 2 Diabetes

**DOI:** 10.3389/fnagi.2022.853695

**Published:** 2022-08-30

**Authors:** Sigalit B. Manzali, Eric Yu, Ramit Ravona-Springer, Abigail Livny, Sapir Golan, Yuxia Ouyang, Orit Lesman-Segev, Lang Liu, Ithamar Ganmore, Anna Alkelai, Ziv Gan-Or, Hung-Mo Lin, Anthony Heymann, Michal Schnaider Beeri, Lior Greenbaum

**Affiliations:** ^1^Department of Pathology, Sheba Medical Center, Tel Hashomer, Israel; ^2^The Joseph Sagol Neuroscience Center, Sheba Medical Center, Tel Hashomer, Israel; ^3^Department of Human Genetics, McGill University, Montréal, QC, Canada; ^4^Montreal Neurological Institute, McGill University, Montréal, QC, Canada; ^5^Memory Clinic, Sheba Medical Center, Tel Hashomer, Israel; ^6^Sackler Faculty of Medicine, Tel Aviv University, Tel Aviv, Israel; ^7^Department of Diagnostic Imaging, Sheba Medical Center, Tel Hashomer, Israel; ^8^Department of Population Health Science and Policy, Icahn School of Medicine at Mount Sinai, New York, NY, United States; ^9^Institute for Genomic Medicine, Columbia University Medical Center, New York, NY, United States; ^10^Department of Neurology and Neurosurgery, McGill University, Montréal, QC, Canada; ^11^Maccabi Healthcare Services, Tel Aviv, Israel; ^12^Department of Psychiatry, Icahn School of Medicine at Mount Sinai, New York, NY, United States; ^13^The Danek Gertner Institute of Human Genetics, Sheba Medical Center, Tel Hashomer, Israel

**Keywords:** Alzheimer’s disease, polygenic risk score, type 2 diabetes, cognitive decline, aging

## Abstract

**Objectives:**

Multiple risk loci for late-onset Alzheimer’s disease (LOAD) have been identified. Type 2 diabetes (T2D) is a risk factor for cognitive decline, dementia and Alzheimer’s disease (AD). We investigated the association of polygenic risk score (PRS) for LOAD with overall cognitive functioning and longitudinal decline, among older adults with T2D.

**Methods:**

The study included 1046 Jewish participants from the Israel Diabetes and Cognitive Decline (IDCD) study, aged ≥ 65 years, diagnosed with T2D, and cognitively normal at baseline. The PRS included variants from 26 LOAD associated loci (at genome-wide significance level), and was calculated with and without *APOE.* Outcome measures, assessed in 18 months intervals, were global cognition and the specific domains of episodic memory, attention/working memory, executive functions, and language/semantic categorization. Random coefficient models were used for analysis, adjusting for demographic variables, T2D-related characteristics, and cardiovascular factors. Additionally, in a subsample of 202 individuals, we analyzed the association of PRS with the volumes of total gray matter, frontal lobe, hippocampus, amygdala, and white matter hyperintensities. Last, the association of PRS with amyloid beta (Aβ) burden was examined in 44 participants who underwent an ^18^F-flutemetamol PET scan.

**Results:**

The PRS was not significantly associated with overall functioning or decline in global cognition or any of the specific cognitive domains. Similarly, following correction for multiple testing, there was no association with Aβ burden and other brain imaging phenotypes.

**Conclusion:**

Our results suggest that the cumulative effect of LOAD susceptibility loci is not associated with a greater rate of cognitive decline in older adults with T2D, and other pathways may underlie this link.

## Introduction

Type 2 diabetes (T2D) is considered as an established risk factor for cognitive impairment, dementia, and Alzheimer’s disease (AD) (McCrimmon et al., 2012; Koekkoek et al., 2015; Zhang et al., 2017). Several mechanisms may contribute to this association, including brain insulin resistance, cerebrovascular disease, inflammation, reduced blood-brain barrier integrity, accumulation of advanced glycation end products, and others (Beeri et al., 2009; Biessels and Despa, 2018). Although brain insulin resistance influences amyloid and tau pathology (Guerrero-Berroa et al., 2014; Berlanga-Acosta et al., 2020; Kellar and Craft, 2020), the cerebral burden of AD-related pathology is not increased in individuals with T2D compared to non-diabetic (Beeri et al., 2005; Arvanitakis et al., 2006; Abner et al., 2016; Dos Santos Matioli et al., 2017; Biessels et al., 2020; Kellar and Craft, 2020).

Multiple late-onset AD (LOAD) susceptibility loci have been identified and replicated, mainly by implementing genome-wide association studies (GWAS) approach. The effect size of these loci is usually small, but is larger for few such as *TREM2* or *APOE*, and *APOE* ε4 allele is the strongest genetic risk factor for LOAD (Karch and Goate, 2015; Naj et al., 2017; Andrews et al., 2020; Bellenguez et al., 2020; Neuner et al., 2020). In addition to analysis of single variants, using of polygenic risk score (PRS), which sums the weighted risk allele count of numerous variants, is an alternative tool for detecting associations with phenotypes of interest (Lambert et al., 2019; Lewis and Vassos, 2020). The number of genetic variants, usually single nucleotide polymorphisms (SNPs), included in PRS calculation differs between studies, and depends on methodological considerations. Some studies calculate PRS by using only a limited number of variants that reached GWAS significance level, while others apply a *p*-value threshold approach, that may include up to many thousands of variants (Harrison et al., 2020; Leonenko et al., 2021).

AD PRS has been associated with a wide range of phenotypes, among them prediction the risk of AD and mild cognitive impairment (MCI), cognitive functioning (both for global or specific domains, such as memory), brain magnetic resonance imaging (MRI) volumetric findings, brain amyloid beta (Aβ) burden measured by positron emission tomography (PET), or amyloid and tau biomarkers in the cerebrospinal fluid (reviewed by Chasioti et al., 2019; Harrison et al., 2020; Zhou et al., 2021).

The main goal of the current study was to investigate the potential effect of LOAD PRS on cognitive decline in older Jewish adults with T2D. The PRS was composed of genetic variants from LOAD associated loci (at genome-wide significance level) in individuals of European ancestry, taken from the studies of Lambert et al. (2013) and Kunkle et al. (2019). Since T2D-related characteristics (like glycemic control) or cardiovascular factors may affect the associations, their interactions with the PRS were also tested. In a subsample of participants, we examined the association of PRS with volumes of total gray matter, frontal lobe, hippocampus, amygdala, and white matter hyperintensities (WMH) or Aβ burden. We hypothesized that a higher PRS would be associated with accelerated cognitive decline, smaller brain region volumes and higher WMH volume and Aβ burden.

## Methods

### Participants

The study population is based on the Israel Diabetes and Cognitive Decline (IDCD) study, comprehensively described by Beeri et al. (2014). Briefly, the study enrolls T2D participants living in the central part of Israel, aged ≥ 65 years, from the diabetes registry of Maccabi Healthcare Services (MHS), the second-largest health maintenance organization in Israel. The subjects underwent a comprehensive cognitive battery and functional assessment, including the clinical dementia rating (CDR) scale (Morris, 1993). Subjects’ cognitive status at baseline (normal, MCI, or dementia) was defined by a multidisciplinary team. Only cognitively normal individuals at baseline, with a CDR score of zero, were eligible to participate in the IDCD study. All were fluent in Hebrew and with no major medical, psychiatric, or neurological conditions that may affect cognitive performance.

The MHS diabetes registry has detailed information on diagnoses, medication, and laboratory results. Further details and description of entry criteria for the registry and eligibility for the IDCD, have been provided in previous reports (Ravona-Springer et al., 2013, 2014; Beeri et al., 2014).

### Cognitive Assessment

All participants underwent a neuropsychological assessments at baseline. When possible, follow-up assessments were administrated at intervals of approximately 18 months. The neuropsychological tests were grouped into 4 cognitive domains, as previously described (Beeri et al., 2014): (1) Episodic memory (AD assessment scale word list immediate recall, delayed recall, and recognition); (2) Attention/working memory (diamond cancellation, digit span forward, and backward); (3) Executive functions (trails making test A and B, and digit symbol substitution test); and (4) Language/semantic categorization (letter and category fluency, and similarities). Each cognitive test score was converted to a *z*-score, and normalized based on the corresponding baseline mean and standard deviation. The *z*-scores of the tests within a domain were first averaged and then normalized again by its mean and standard deviation to create a domain specific composite score. Global cognition *z*-score was obtained by final normalization of the averaged domain *z*-scores.

### Selection of Genetic Variants, Genotyping, and Polygenic Risk Score Calculation

Following two large scale GWAS meta-analyses of LOAD (Lambert et al., 2013; Kunkle et al., 2019) in individuals of European ancestry, we genotyped the lead variant in each LOAD loci that reached genome-wide significance level in at least one study (in addition to *APOE* variants, as detailed below). For loci that were significant in both studies, the lead variant (with the lowest *p*-value) was genotyped according to Lambert et al. (2013). As part of the International Genomics of Alzheimer’s disease Project (IGAP), these meta-analyses of 74,046 and 94,437 participants, respectively, confirmed previous associations and discovered novel ones.

Based on Lambert et al. (2013) study, the variants were rs6656401 (*CR1*), rs6733839 (*BIN1*), rs35349669 (*INPP5D)*, rs190982 (*MEF2C*), rs9271192 (*HLA-DRB5- HLA-DRB1*), rs10948363 (*CD2AP*), rs2718058 (*NME8*), rs11771145 (*EPHA1*), rs1476679 (*ZCWPW1)*, rs28834970 (*PTK2B*), rs9331896 (*CLU*), rs10838725 (*CELF1*), rs983392 (*MS4A6A*), rs10792832 (*PICALM*), rs11218343 (*SORL1*), rs17125944 (*FERMT2*), rs10498633 (*SLC24A4 RIN3)*, rs4147929 (*ABCA7*), and rs7274581 (*CASS4).* Although *MEF2C* and *NME8* loci associations were not further replicated by Kunkle et al. (2019) and deserve further study, we still incorporated them in the PRS calculation.

From Kunkle et al. (2019) study, we took rs75932628 (*TREM2*), rs7920721 (*ECHDC3*), rs593742 (*ADAM10*), rs7185636 (*IQC*K), rs138190086 (*ACE*), rs62039712 (*WWOX*), and rs2830500 (*ADAMTS1*). The PRS included only the lead variant for each of the abovementioned loci, although some loci may harbor additional independent LOAD association signals (for example, in the *TREM2* region).

Last, variants rs7412 and rs429358 were genotyped to determine the *APOE* status (*APOE* ε2, ε3, and ε4 alleles).

Genomic DNA was extracted from blood. The variants were genotyped using Kompetitive Allele-Specific PCR (KASP) technology by LGC Genomics (Teddington, United Kingdom). About 10% of the sample was blindly double-genotyped for quality control purposes, and the concordance rate between duplicates was above 99%.

PRS was calculated using PRSice-2 v2.3.3^1^ (Choi and O’Reilly, 2019) as a summation of genotyped variants (allele dosage) weighted by effect size, based on the relevant Lambert et al. (2013) or Kunkle et al. (2019) meta-analyses data. All available genetic variants were included in the PRS, which was calculated with and without the *APOE* variants rs7412 and rs429358. Then, the PRS (with and without *APOE*) was z-transformed for further analysis.

### Magnetic Resonance Imaging Acquisition

A subsample of the study participants underwent a brain MRI scan, performed by a 3 Tesla scanner (GE, Signa HDxt, v16VO2). MRI acquisition and analysis methods are described in details elsewhere (Livny et al., 2016; Ganmore et al., 2019). In brief, a 3D inversion recovery prepared fast spoiled gradient-echo (FSPGR) T1-weighted sequence and a T2-weighted fluid-attenuated inversion recovery (FLAIR) sequence were acquired. For volumetric analysis, the voxel based morphometry (VBM) toolbox^2^ (Ashburner and Friston, 2000), implemented in Statistical Parametric Mapping (SPM8) software, was used. Focusing on MRI phenotypes that are related to AD, neurodegeneration, or brain aging, we examined total gray matter volume and applied a region of interest (ROI) approach centered on the total regional volume of frontal lobe, hippocampus, and amygdala. For WMH quantification, we used the lesion segmentation toolbox (LST), implemented in SPM8 (Livny et al., 2016).

### ^18^F-flutemetamol Aβ Positron Emission Tomography Acquisition and Preprocessing

^18^F-flutemetamol (Vizamyl, GE Healthcare) was synthesized at the Hadassah Medical Center cyclotron radiochemistry unit. PET scans were performed for part of the MRI subsample on a Philips Vereos PET/computered tomography (CT) scanner in 3D acquisition mode, at Sheba Medical Center. Prior to all scans, a low-dose CT scan was conducted for attenuation correction. Image acquisition began 90 min post-injection of 4–5 millicuries and took 20 min. Iterative reconstruction with weighted attenuation scatter was performed with a slice thickness of 2 mm, matrix size of 128 × 128 with pixel sizes of 2 × 2 mm.

Each patient’s closest T1 weighted MRI was segmented by FreeSurfer 6.0^3^ and SPM12 software to define the reference region. PET frames were then co-registered onto their corresponding MRI by SPM12 and standardized uptake value ratio (SUVR) maps were calculated with FreeSurfer-defined whole cerebellum as a reference region. The global cortical uptake value was then extracted in native space using large, Freesurfer-defined cortical ROIs. This value was used as a measure of Aβ burden. Further information on ^18^F-flutemetamol Aβ PET methods is provided by Ravona-Springer et al. (2020).

### Statistical Analysis

#### Polygenic Risk Score and Cognition

For global cognition and the specific domains, we aimed to study the association of PRS with overall cognitive functioning (for all available measurements, from baseline and through the whole follow-up period), and with longitudinal cognitive decline. Random coefficient models were used to describe the trend of cognitive *z*-score over the follow-up period. As in our former studies (Ganmore et al., 2020; Ravona-Springer et al., 2021; Soleimani et al., 2021), we assumed a linear trajectory of the cognitive data, and that the coefficients of intercept and slope are unique to each participant.

The models included the participant PRS, time (in months), and the PRS by time interaction. This allowed evaluation of the PRS effect over time. For example, a significant negative interaction between the PRS and time would suggest that for individuals with a high PRS, the overall cognitive decline was greater over time than for individuals with a low PRS. Each analysis was performed twice, for PRS with and without the *APOE* locus. The analysis was then repeated by using reduced models that removed the PRS interaction with time.

The first model (model 1) was adjusted for the following demographic variables: age at baseline, sex, years of education, and ancestry (Ashkenazi vs. Non-Ashkenazi Jewish descent, based on self-report). In the second model (model 2), the analysis was repeated while adjusting also for T2D-related characteristics (hemoglobin A1c [HbA1C] and duration in the MHS diabetes registry, which is a proxy for T2D duration; West et al., 2014) and cardiovascular factors (systolic and diastolic blood pressure, total cholesterol, triglycerides, creatinine and body mass index [BMI]). Finally, we additionally adjusted for physical activity index, determined by the number of various physical activities performed by the subject over the previous 2 weeks. This was assessed by a simplified version of the Minnesota Leisure Time Activity Questionnaire (Taylor et al., 1978).

In a secondary analysis, we investigated the potential effect of the T2D-related characteristics on the association between PRS and cognitive decline, for global cognition and the specific domains. Using the same covariates as in models 1 and 2, we examined the interactions of the HbA1c or duration in the T2D registry terms with PRS and with time (three-way interaction). A similar analysis was performed for the cardiovascular factors.

In the absence of a significant three-way interaction effect (following correction for multiple testing), we repeated the analyses, investigating the effect of the two-way interaction of PRS with T2D-related characteristics or cardiovascular factors, on overall cognitive functioning.

#### Polygenic Risk Score and Brain Imaging Data

We employed linear regression models to study the association of the PRS with total gray matter, frontal lobe, hippocampus, amygdala, and WMH volumes and with Aβ burden. Each subsample participant underwent a single brain MRI and some also had a single PET imaging, and this analysis was therefore cross-sectional. Model 1 was adjusted for demographics and total intra-cranial volume (TICV); however, TICV was not included in the models for WMH and Aβ burden. Model 2 had all these covariates, in addition to the set of T2D-related characteristics and cardiovascular factors. Again, the analysis was performed twice, for PRS with and without *APOE*.

SAS 9.4 software (SAS Institute, Cary, NC, United States) was used to conduct statistical analysis. All *p*-values were two-sided. For the cognitive analysis and the interaction study, the *p*-value threshold for multiple testing significance level was 0.01 [0.05 divided by the number of outcomes measures (*N* = 5)]. For the brain imaging phenotypes, it was 0.0083 (0.05/6 outcomes).

## Results

The final sample included 1046 Jewish IDCD participants with demographic data, PRS, and cognitive assessments. Among them, 805 (77.0%) had at least one follow-up assessment, 617 (59.0%) had at least two, and 198 (18.9%) had three or more follow-up assessments, at intervals of approximately 18 months.

The mean age at study baseline was 72.5 ± 4.8 years, 631 (60.3%) of participants were males, and the mean years of education was 13.0 ± 3.6. The median follow-up time was 36 months (interquartile range 24 – 54). At baseline, 714 (68.3%), participants used antidiabetic medication (hypoglycemic medication and/or insulin), 135 (12.9%) did not take antidiabetic medication and this information was missing for 197 (18.8%). The MRI and PET imaging were performed during the follow-up period, on average 4.3 ± 1.0 years after baseline cognitive assessment for the MRI (*N* = 202), and 7.6 ± 0.8 years for PET (*N* = 44). Overtime, the subjects’ decline in global cognition, illustrated in Supplementary Figure 1, was significant (annual slope of *z*-score change: –0.098, *SE* = 0.0037, *p* < .001). Further description of the sample is provided in Table 1.

**TABLE 1:** Demographic and clinical characteristics [mean (SD) unless otherwise indicated] of the sample: cognitive measurements, brain MRI, and Aβ PET.

Variable	Cognitive measurements	Brain MRI	Aβ PET
*N*	1046	202	44
Age at IDCD study baseline, years	72.5 (4.8)	71.2 (4.3)	70.8 (3.4)
Male sex, *N*,%	631 (60.3%)	126 (62.4%)	30 (68.2%)
Years of education	13.0 (3.6)	13.8 (3.5)	14.0 (3.9)
Ashkenazi ancestry, *N*,%	503 (48.1%)	127 (62.9%)	25 (56.8%)
HbA1c (%)	6.8 (0.8)	6.7 (0.8)	6.6 (1.1)
Duration in the T2D registry at IDCD study baseline, years	9.7 (4.4)	9.2 (4.5)	8.9 (4.7)
Systolic blood pressure (mmHg)	134.7 (8.6)	133.8 (8.2)	134.3 (8.9)
Diastolic blood pressure (mmHg)	75.7 (4.7)	76.0 (4.8)	76.2 (4.8)
Total cholesterol (mg/dL)	174.2 (24.5)	173.9 (23.5)	169.9 (25.2)
Triglycerides (mg/dL)	156.0 (61.8)	150.8 (73.9)	148.7 (54.5)
Creatinine (mg/dL)	1.0 (0.3)	0.98 (0.2)	0.99 (0.3)
BMI (kg/m^2^)	28.6 (4.2)	28.5 (4.5)	28.8 (4.4)

*The HbA1c and cardiovascular factors are means of all historical measurements in the MHS registry until study enrollment, and were available only for 841 individuals. Aβ, amyloid beta; BMI, body mass index; HbA1c, hemoglobin A1c; IDCD, Israel Diabetes and Cognitive decline; MHS, Maccabi Healthcare System; MRI, magnetic resonance imaging; N, number; PET, positron emission tomography; SD, standard deviation; T2D, type 2 diabetes.*

The variant rs62039712 (*WWOX*) failed genotyping. All other variants were successfully genotyped with calling rate >98% for each, and included in the PRS calculation (Supplementary Table 1). None showed a deviation from the Hardy-Weinberg equilibrium (*p* > 0.05). In total, the PRS with and without *APOE* included 25 and 27 genetic variants, respectively. The frequency of the *APOE* genotypes was as follow: ε2/ε2-0.86%, ε2/ε3-12.72%, ε2/ε4-1.43%, ε3/ε3-72.37%, ε3/ε4-11.95%, ε4/ε4- 0.67%. There were no significant differences in the mean PRS between participants with only baseline data and participants who had at least one follow-up assessment (*p* = 0.62 and *p* = 0.19 for PRS with and without *APOE*, accordingly).

As presented in Table 2, there were no significant associations of the PRS for LOAD (with and without *APOE*) with baseline overall functioning or longitudinal decline, in global cognition or any of the specific cognitive domains (model 1, *N* = 1046). Similar results were seen when the analyses were adjusted also for T2D-related characteristics and cardiovascular factors (model 2, *N* = 841). Likewise, the effect of PRS on overall cognitive functioning was non-significant in the reduced models that removed the PRS interaction with time (Supplementary Table 2). We then incorporated the physical activity index (mean 3.5 ± 2.2) as an additional covariate in model 2, and results were essentially unchanged (data not shown).

**TABLE 2:** Association of the PRS with overall cognitive functioning and with longitudinal cognitive decline.

		Model 1						Model 2					
		Without *APOE*			*With APOE*			Without *APOE*			With *APOE*		
Cognitive domain	Effect #	Estimate	*SE*	*p*	Estimate	*SE*	*p*	Estimate	*SE*	*p*	Estimate	*SE*	*p*
Global cognition	PRS	0.0093	0.0242	0.700	–0.0004	0.0241	0.985	0.0072	0.0243	0.769	0.0084	0.0236	0.722
	PRS * t	0.0002	0.0003	0.442	–0.0001	0.0003	0.802	0.0004	0.0003	0.248	–0.0003	0.0003	0.367
Episodic memory	PRS	–0.0188	0.0259	0.468	–0.0322	0.0258	0.213	–0.0246	0.0245	0.315	–0.0413	0.0237	0.082
	PRS * t	0.0002	0.0005	0.678	–0.0001	0.0005	0.919	0.0005	0.0005	0.390	0.00003	0.0005	0.952
Attention/Working memory	PRS	0.0049	0.0266	0.854	0.0071	0.0265	0.790	0.0062	0.0287	0.830	0.0108	0.0279	0.700
	PRS * t	0.0005	0.0004	0.234	0.0003	0.0004	0.520	0.0006	0.0005	0.194	–0.00002	0.0005	0.961
Executive functions	PRS	0.0159	0.0245	0.517	0.0183	0.0245	0.456	0.0297	0.0257	0.249	0.0420	0.0251	0.094
	PRS * t	0.0002	0.0004	0.613	–0.0003	0.0004	0.378	0.0002	0.0004	0.615	–0.0007	0.0004	0.103
Language/semantic categorization	PRS	0.0181	0.0265	0.495	0.0103	0.0264	0.697	0.0130	0.0281	0.645	0.0149	0.0274	0.587
	PRS * t	0.0003	0.0003	0.352	0.00004	0.0003	0.889	0.0002	0.0003	0.542	–0.0002	0.0003	0.581

# PRS effect refers to baseline cognitive functioning; PRS * t effect refers to cognitive decline over time (months).

*Model 1 (N = 1046): adjusted for sex, age, years of education, and ancestry.*

*Model 2 (N = 841): adjusted also for HbA1c, duration in the T2D registry, systolic and diastolic blood pressure, total cholesterol, triglycerides, creatinine, and BMI.*

*BMI, body mass index; HbA1c, hemoglobin A1c; PRS, polygenic risk Score; SE, standard error; t, time; T2D, type 2 diabetes.*

In the secondary analysis (*N* = 841), the three-way interactions of T2D-related characteristics (HbA1c and duration in the diabetes registry) or cardiovascular factors (systolic and diastolic blood pressure, total cholesterol, triglyceride, creatinine, and BMI) with PRS were not significantly associated with the rate of cognitive decline, following correction for multiple testing. Then, we removed the three-way interaction terms and repeated the analyses. Similarly, the two-way interactions of PRS with these T2D-related characteristics or cardiovascular factors had no significant associations with overall cognitive functioning. The nominal level (0.01 < *p* < 0.05) PRS interactions, mainly with diastolic blood pressure (two- and three-ways), are presented in Supplementary Tables 3, 4.

For the neuroimaging phenotypes, the PRS (with and without *APOE*) was not significantly associated with total gray matter volume, or with volumes of the frontal lobe, hippocampus, amygdala, and WMH, nor with Aβ burden (Table 3), after correction for multiple testing. Surprisingly, contrary to our hypothesis, larger amygdala and frontal lobe volumes were nominally associated with higher PRS (excluding *APOE*): *p* = 0.036 for the amygdala and *p* = 0.05 for frontal lobe in model 1; *p* = 0.072 and *p* = 0.033 in model 2, respectively. However, these results did not withstand adjustment for multiple comparison, and when *APOE* was included in the PRS, the associations were non-significant (all *p* ≥ 0.609).

**TABLE 3:** Association of the PRS with brain MRI phenotypes (volume of gray matter, frontal lobe, hippocampus, amygdala, and WMH) and with Aβ burden.

	Model 1						Model 2					
	Without *APOE*			*With APOE*			Without *APOE*			With *APOE*		
Imaging	Estimate	*SE*	*p*	Estimate	*SE*	*p*	Estimate	*SE*	*p*	Estimate	*SE*	*p*
Gray matter	0.6151	2.0969	0.770	–1.7470	1.7940	0.331	0.8502	2.1638	0.695	–1.2904	1.8697	0.491
Frontal lobe	0.0041	0.0021	0.050	0.0001	0.0018	0.969	0.0047	0.0022	0.033	–0.0002	0.0019	0.898
Hippocampus	0.0002	0.0031	0.951	–0.0039	0.0026	0.142	–0.0003	0.0033	0.939	–0.0043	0.0028	0.123
Amygdala	0.0130	0.0062	0.036	–0.0027	0.0053	0.613	0.0115	0.0064	0.072	–0.0029	0.0056	0.609
WMH	0.2015	1.0666	0.850	0.0898	0.9056	0.921	–0.0044	1.1581	0.997	0.2183	0.9928	0.826
Aβ burden*	–0.0008	0.0412	0.985	0.0199	0.0367	0.590	0.0057	0.0482	0.906	0.0491	0.0441	0.275

*Model 1 (N = 202; N = 44 for Aβ burden): adjusted for sex, age at imaging time, years of education and ancestry. TICV was also adjusted for gray matter, frontal lobe, hippocampus, and amygdala.*

*Model 2 (N = 188; N = 43 for Aβ burden): adjusted also for HbA1c, duration in the T2D registry, systolic and diastolic blood pressure, total cholesterol, triglyceride, creatinine, and BMI.*

*Aβ, amyloid beta; BMI, body mass index; HbA1c, hemoglobin A1c; MRI, magnetic resonance imaging; SE, standard error; TICV, total intracranial volume; T2D, type 2 diabetes; WMH, white matter hyperintensities.*

*Aβ global cortical uptake value.

## Discussion

There is robust evidence for the link between T2D and dementia risk, including AD. In this study, we were unable to show a significant association of LOAD PRS with overall functioning or longitudinal decline in global cognition and specific cognitive domains, in a sample of T2D older adults. Moreover, the interactions of the PRS with T2D-related characteristics or cardiovascular factors were not significantly associated with cognitive functioning, following multiple testing correction. Similarly, the PRS was not associated with volume of hippocampus and other brain regions related to AD and various aspects of brain aging, nor with WMH volume and Aβ burden.

Previous studies examined the association of AD PRS with a variety of cognitive phenotypes. Significant associations were found in many but not in all studies, and in some cases the association was mainly dependent on inclusion of the *APOE* locus (e.g., Verhaaren et al., 2013; Harris et al., 2014; Carrasquillo et al., 2015; Marden et al., 2016; Mormino et al., 2016; Andrews et al., 2017; Bressler et al., 2017; Del-Aguila et al., 2018; Tank et al., 2022). In addition, significant associations of AD PRS with hippocampal volume, cortical thickness, PET Aβ burden, and other brain imaging phenotypes were reported (e.g., Sabuncu et al., 2012; Lupton et al., 2016; Mormino et al., 2016; Foley et al., 2017; Foo et al., 2020; Tank et al., 2022). Notably, these analyses were performed in different cohorts, with variable and heterogeneous population characteristics, age range, and methodology. However, we believe our study is the first to investigate the effect of AD PRS specifically in a sample of well-characterized T2D older adults, studying both cognitive functioning and brain imaging measurements. At the single variant analysis level, a previous study in the IDCD cohort investigated the association of 19 LOAD loci (included in the current PRS calculation) with episodic memory performance at baseline, and found a significant association of the *BIN1* variant rs6733839 (Greenbaum et al., 2016).

The IDCD cohort does not include a control group of older adult without T2D. This means we were not able to generalize the findings and determine if the negative results are specific to T2D individuals. Numerous longitudinal studies have compared subjects with and without T2D, and found an accelerated rate of decline in global cognition or other specific cognitive domains in T2D subjects (Nooyens et al., 2010; Yaffe et al., 2012; Spauwen et al., 2013; Tuligenga et al., 2014; Palta et al., 2017) but not in the oldest old population (van den Berg et al., 2006). Our findings suggest that even if there is a faster rate of cognitive decline among T2D patients, we did not observe an association of the PRS with the rate of decline.

A shared genetic etiology between AD and T2D has been suggested (Hao et al., 2015; Gao et al., 2016; Wang et al., 2017). Our results may indicate that cognitive decline in T2D is not affected by the cumulative genetic susceptibility to LOAD. Cognitive impairment in T2D is likely related to mixed pathologies and multiple mechanisms (Beeri et al., 2009; Biessels and Despa, 2018). The current findings corroborate observations regarding the lack of associations between AD-related pathology and T2D, as no increased cerebral AD pathology was shown in T2D patients compared to controls (Beeri et al., 2005; Arvanitakis et al., 2006; Abner et al., 2016; Dos Santos Matioli et al., 2017; Berlanga-Acosta et al., 2020; Biessels et al., 2020; Kellar and Craft, 2020). On the other hand, some of these papers reported higher cerebrovascular pathology in T2D. Although still speculative and requiring validation in additional samples, the genetic architecture underlying cognitive decline in T2D may be less relevant to LOAD, and attributed instead to other mechanisms, such as cerebrovascular disease. Interestingly, the LOAD loci included in our PRS harbour genes that are related to processes and pathways like cholesterol metabolism, immune response, and endocytosis (Karch and Goate, 2015; Kunkle et al., 2019).

The current study population was of Jewish descent, but since LOAD GWAS data specific for this population was unavailable, we calculated the PRS based on data from genome-wide association meta-analyses performed in samples of European ancestry individuals. This is a limitation, since the portability of PRS between populations depends on their genetic distance (Privé et al., 2022). The portability is affected by factors such as differences in linkage disequilibrium patterns, allele frequencies and effect size of associated variants (Grinde et al., 2019; Privé et al., 2022). This may have reduced the accuracy of our analysis, and the results should be therefore interpreted with caution. Generally, as shown by principle component (PC) and population structure analyses, the genetic architecture of the majority of Jewish people is composed of admixture of Middle Eastern, European and other hosting populations at various levels (Behar et al., 2010; Ostrer and Skorecki, 2013). Importantly in this regard, the minor alleles of all 25 lead variants included in the PRS are consistent between the relevant LOAD meta-analysis and our IDCD sample, and their frequencies are generally comparable (Supplementary Table 1). Although we added self-reported ancestry (Ashkenazi vs. Non-Ashkenazi) as a covariate in the statistical model, a better accounting for population stratification in the IDCD cohort (such as using PC analysis) would have been preferable.

Several other limitations of this study should be acknowledged. First, the sample included 1046 participants for the cognitive analysis, 202 with brain MRI data and only 44 for the Aβ PET analysis. Therefore, the lack of significant results may stem from a low statistical power. Second, only 805 participants had cognitive follow-up data, and the follow-up was relatively short for many (median of 36 months). Associations may become visible as the longitudinal component of the IDCD grows. In addition, the MRI and PET were not performed at baseline but at mean of 4.3 and 7.6 years later (respectively), and this time interval may have affected the findings of the cross-sectional imaging studies. Nevertheless, although brain imaging was performed when cognitive decline was already affecting some participants, there were no significant associations with PRS.

We constructed the PRS by the inclusion of lead variants from only 25 loci plus *APOE*, all associated with LOAD at genome-wide significance level (p < 5×10^−8^). However, while most loci were significant in the two meta-analyses, several were associated with LOAD in only one of them. In addition, for some loci, two independent association signals may exist rather than one, thus underestimating their contribution to PRS. Moreover, additional risk loci were reported in recent genome-wide meta-analyses that included also AD-by-proxy cases and controls (Jansen et al., 2019; de Rojas et al., 2021; Schwartzentruber et al., 2021; Wightman et al., 2021). Thus, the updated number of LOAD risk loci is higher than included in our PRS, and rapidly expands. Of note, different approaches for PRS calculation are based on inclusion of very large numbers of variants with lower *p*-value thresholds of association with the phenotype, based on GWAS data. Unfortunately, GWAS genotyping is not available for the IDCD sample.

The present study is unique, and combines genetic data, comprehensive cognitive assessments over time, brain MRI and Aβ PET phenotypes, and a broad range of relevant and directly measured (rather than self-reported) clinical information. Since the effect of genetic susceptibility is probably influenced by additional risk factors, we specifically examined the influence of interaction between PRS and T2D-related characteristics or cardiovascular factors on cognitive measures. Interestingly, a former study in the IDCD cohort showed that *APOE* ε*4* status modified the relationship of long-term glycemic control with cognitive functioning (Ravona-Springer et al., 2014). However, none of the interactions of the PRS with T2D-related characteristics or cardiovascular factors were significant, following adjustment for multiple testing. Several associations at nominal level (0.01 < *p* < 0.05) were observed, mainly for the interactions of PRS with diastolic blood pressure (two- and three-ways), and future studies in larger samples with longer follow-up may clarify these findings.

To conclude, we did not find a significant association of LOAD PRS with overall cognitive functioning or rate of decline in T2D older adults. The negative results may indicate that brain pathophysiology underlying the link between T2D and cognitive decline is not influenced by the genetic underpinnings of LOAD, and investigation of additional pathways is required.

## Data Availability Statement

The raw data supporting the conclusions of this article will be made available by the corresponding author on reasonable request.

## Ethics Statement

This study was reviewed and approved by the institutional review boards of Sheba Medical Center and MHS, Israel, and Icahn school of Medicine at Mount Sinai, NY. The participants provided written informed consent.

## Author Contributions

SBM, MSB, and LG designed the study, researched the data, and wrote the manuscript. EY and LL contributed to the genetic analysis and interpretation of data. YO and H-ML contributed to the statistical analysis and interpretation of data. AL, SG, and OL-S acquired neuroimaging data and revised the manuscript. RR-S, IG, AA, ZG-O, and AH took part in the interpretation of data and revised the manuscript. All authors approved the submitted version.

## Conflict of Interest

The authors declare that the research was conducted in the absence of any commercial or financial relationships that could be construed as a potential conflict of interest.

## Publisher’s Note

All claims expressed in this article are solely those of the authors and do not necessarily represent those of their affiliated organizations, or those of the publisher, the editors and the reviewers. Any product that may be evaluated in this article, or claim that may be made by its manufacturer, is not guaranteed or endorsed by the publisher.

## Footnotes

1
https://www.prsice.info/

2
http://www.fil.ion.ucl.ac.uk/spm/ext/#VBMtools

3
https://surfer.nmr.mgh.harvard.edu/
