# Supplementary material for: Alzheimer’s Disease Polygenic Risk Score Is Not Associated With Cognitive Decline Among Older Adults With Type 2 Diabetes
Source: Front Aging Neurosci. 2022 Aug 30;14:853695. doi: 10.3389/fnagi.2022.853695 (PMC9468264; doi:10.3389/fnagi.2022.853695)
Supplement: Supplementary file 1 [file Data_Sheet_1.pdf]

Supplementary Table 1: List of variants included in the PRS calculation (without *APOE*)

Variant	Chr.	Closest gene	Major/Minor allele	Present study MAF	Previously reported MAF*	Previously reported OR (95% CI)*	Reference
rs6656401	1	*CR1*	G/A	0.176	0.197	1.18 (1.14-1.22)	1
rs6733839	2	*BIN1*	C/T	0.342	0.409	1.22 (1.18-1.25)	1
rs35349669	2	*INPP5D*	C/T	0.365	0.488	1.08 (1.05-1.11)	1
rs190982	5	*MEF2C*	A/G	0.454	0.408	0.93 (0.90-0.95)	1
rs9271192	6	*HLA-DRB5- HLA-DRB1*	A/C	0.165	0.276	1.11 (1.08-1.15)	1
rs75932628	6	*TREM2*	C/T	0.006	0.008	2.08 (1.73-2.49)	2
rs10948363	6	*CD2AP*	A/G	0.215	0.266	1.10 (1.07-1.13)	1
rs2718058	7	*NME8*	A/G	0.371	0.373	0.93 (0.90-0.95)	1
rs11771145	7	*EPHA1*	G/A	0.303	0.338	0.90 (0.88-0.93)	1
rs1476679	7	*ZCWPW1***	T/C	0.239	0.287	0.91 (0.89-0.94)	1
rs28834970	8	*PTK2B*	T/C	0.300	0.366	1.10 (1.08-1.13)	1
rs9331896	8	*CLU*	T/C	0.394	0.379	0.86 (0.84-0.89)	1
rs7920721	10	*ECHDC3*	A/G	0.408	0.390	1.08 (1.06-1.11)	2
rs10838725	11	*CELF1***	T/C	0.305	0.316	1.08 (1.05-1.11)	1
rs983392	11	*MS4A6A*	A/G	0.430	0.403	0.90 (0.87-0.92)	1
rs10792832	11	*PICALM*	G/A	0.393	0.358	0.87 (0.85-0.89)	1
rs11218343	11	*SORL1*	T/C	0.053	0.039	0.77 (0.72-0.82)	1
rs17125944	14	*FERMT2*	T/C	0.055	0.092	1.14 (1.09-1.19)	1
rs10498633	14	*SLC24A4 RIN3*	G/T	0.210	0.217	0.91 (0.88-0.94)	1
rs593742	15	*ADAM10*	A/G	0.266	0.295	0.93 (0.91-0.95)	2
rs7185636	16	*IQCK*	T/C	0.245	0.180	0.92 (0.89-0.95)	2
rs138190086	17	*ACE*	G/A	0.019	0.020	1.30 (1.19-1.42)	2
rs4147929	19	*ABCA7*	G/A	0.166	0.190	1.15 (1.11-1.19)	1
rs7274581	20	*CASS4*	T/C	0.173	0.083	0.88 (0.84-0.92)	1
rs2830500	21	*ADAMTS1*	C/A	0.386	0.308	0.93(0.91-0.96)	2

*Data is derived from Lambert et al. (2013) or Kunkle et al. (2019); MAF is the average in the discovery stage, OR and CI (overall analysis) are calculated with respect to the minor allele. Notably, the MAF data from these meta-analyses is similar to the frequency in gnomAD v.2.1.1 (https://gnomad.broadinstitute.org/) in the European (non-Finnish) population. For all variants, the minor allele is consistent between the present study and the European population.

**The *ZCWPW1* and *CELF1* loci are referred as *NYAP1* and *SPI1*, respectively, by Kunkle et al. (2019).

Chr, chromosome; CI, confidence interval; MAF, minor allele frequency; OR, odds ratio; PRS, polygenic risk score.

References:

1. Lambert, J. C., Ibrahim-Verbaas, C. A., Harold, D., Naj, A. C., Sims, R., Bellenguez, C., et al. (2013). Meta-analysis of 74,046 individuals identifies 11 new susceptibility loci for Alzheimer's disease. Nat. Genet. 45, 1452–1458. doi:10.1038/ng.2802

2. Kunkle, B. W., Grenier-Boley, B., Sims, R., Bis, J. C., Damotte, V., Naj, A. C., et al. (2019). Genetic meta-analysis of diagnosed Alzheimer's disease identifies new risk loci and implicates Ab, tau, immunity and lipid processing. Nat. Genet. 51,414–430. doi: 10.1038/s41588-019-0358-2

Supplementary Table 2: Association of the PRS with overall cognitive functioning (all available measurements)

	Model 1						Model 2					
	Without *APOE*			*With APOE*			Without *APOE*			With *APOE*		
Cognitive Domain	Estimate	SE	p	Estimate	SE	p	Estimate	SE	p	Estimate	SE	p
Global cognition	0.0118	0.0240	0.624	-0.0011	0.0240	0.962	0.0106	0.0242	0.662	0.0062	0.0235	0.791
Episodic memory	-0.0141	0.0233	0.546	-0.0333	0.0234	0.155	-0.0162	0.0225	0.472	-0.0407	0.0220	0.064
Attention / Working memory	0.0136	0.0256	0.594	0.0116	0.0256	0.649	0.0160	0.0276	0.562	0.0104	0.0270	0.700
Executive functions	0.0183	0.0241	0.448	0.0143	0.0241	0.553	0.0319	0.0254	0.209	0.0354	0.0248	0.153
Language / semantic categorization	0.0245	0.0257	0.341	0.0112	0.0256	0.662	0.0178	0.0270	0.509	0.0107	0.0264	0.685

Model 1 (N=1046): adjusted for sex, age, years of education, and ancestry.

Model 2 (N=841): adjusted also for HbA1c, duration in the T2D registry, systolic and diastolic blood pressure, total cholesterol, triglycerides, creatinine, and BMI.

BMI, body mass index; HbA1c, hemoglobin A1c; PRS, polygenic risk score; SE, standard error; T2D, type 2 diabetes.

Supplementary Table 3: Nominally significant 3-way interactions of PRS, time (months), and T2D-related characteristics or cardiovascular factors ($p<0.05$)

As explained in the main text, the 3-way interaction analysis (N=841) evaluates the influence of T2D-related characteristics or cardiovascular factors (means of all measurements in the MHS registry until study enrollment) on the PRS effect on cognition over time. For example, a positive 3-way interaction between PRS, time, and DBP suggests that a higher DBP will slow down the PRS effect on cognitive decline.

None of the interactions withstood correction for multiple testing.

A) PRS without *APOE*

		Model 1			Model 2		
Dependent variable (cognitive domain)	Interaction of PRS * t with	Estimate	SE	p	Estimate	SE	p
Global cognition	DBP	0.00015	0.00007	0.031	0.00015	0.00007	0.031
Episodic memory	DBP	0.00025	0.00011	0.021	0.00025	0.00011	0.022

B) PRS with *APOE*

		Model 1			Model 2		
Dependent variable (cognitive domain)	Interaction of PRS * t with	Estimate	SE	p	Estimate	SE	p
Episodic memory	BMI	-0.00031	0.00014	0.028	-0.00031	0.00014	0.028
Global cognition	DBP	0.00016	0.00007	0.028	0.00016	0.00007	0.027
Language / semantic categorization	DBP	0.00015	0.00007	0.030	0.00016	0.00007	0.028
Episodic memory	Creatinine	-0.00385	0.00194	0.047	-0.00380	0.00194	0.050

Each model included PRS, time, a single T2D-related characteristics or cardiovascular factor, and their interactions.

Model 1: adjusted for sex, age, years of education, and ancestry.

Model 2: adjusted also for additional T2D-related characteristics and cardiovascular factors- HbA1c, duration in the T2D registry, systolic and diastolic blood pressure, total cholesterol, triglyceride, creatinine, and BMI.

BMI, body mass index; DBP, diastolic blood pressure; HbA1c, Hemoglobin A1c; MHS, Maccabi Healthcare Services; PRS, polygenic risk score; SE, standard error; t, time in months; T2D, type 2 diabetes.

Supplementary Table 4: Nominally significant 2-way interactions of PRS and T2D-related characteristics or cardiovascular factors ($p<0.05$)

Since the corresponding higher level of 3-way interaction terms with time were all non-significant after adjusting for multiple comparisons, we removed the 3-way interaction terms and repeated the analyses (N=841), in order to study the 2-way interaction of PRS with T2D-related characteristics or cardiovascular factors. The 2-way interaction refers to the influence of these variables on the PRS effect on overall cognitive functioning. For example, a positive interaction between PRS and DBP suggests that a higher DBP will alleviate the effect of PRS on cognition.

None of the interactions withstood correction for multiple testing.

A) PRS without *APOE*

		Model 1			Model 2		
Dependent variable (cognitive domain)	Interaction of PRS with	Estimate	SE	p	Estimate	SE	p
Global cognition	DBP	0.01144	0.00510	0.025	0.01088	0.00508	0.033
Language / semantic categorization	DBP	0.01288	0.00570	0.024	0.01212	0.00568	0.033
Episodic memory	DBP	0.00956	0.00474	0.044	0.00917	0.00477	0.055

B) PRS with *APOE*

		Model 1			Model 2		
Dependent variable (cognitive domain)	Interaction of PRS with	Estimate	SE	p	Estimate	SE	p
Executive functions	DBP	0.00983	0.00521	0.060	0.01030	0.00516	0.046

Each model included PRS, time, a single T2D-related characteristics or cardiovascular factor and their interactions.

Model 1: adjusted for sex, age, years of education, and ancestry.

Model 2: adjusted also for additional T2D-related characteristics and cardiovascular factors- HbA1c, duration in the T2D registry, systolic and diastolic blood pressure, total cholesterol, triglyceride, creatinine, and BMI.

BMI, body mass index; DBS, diastolic blood pressure; HbA1c, Hemoglobin A1c; PRS, polygenic risk score; SE, standard error; T2D, type 2 diabetes.
