# Supplementary material for: Alzheimer’s Disease Polygenic Risk Score Is Not Associated With Cognitive Decline Among Older Adults With Type 2 Diabetes
Source: Front Aging Neurosci. 2022 Aug 30;14:853695. doi: 10.3389/fnagi.2022.853695 (PMC9468264; doi:10.3389/fnagi.2022.853695)
Supplement: Supplementary file 2 [file Image_1.pdf]

Supplementary Figure 1: Trend of decline in global cognition (z-score) overtime

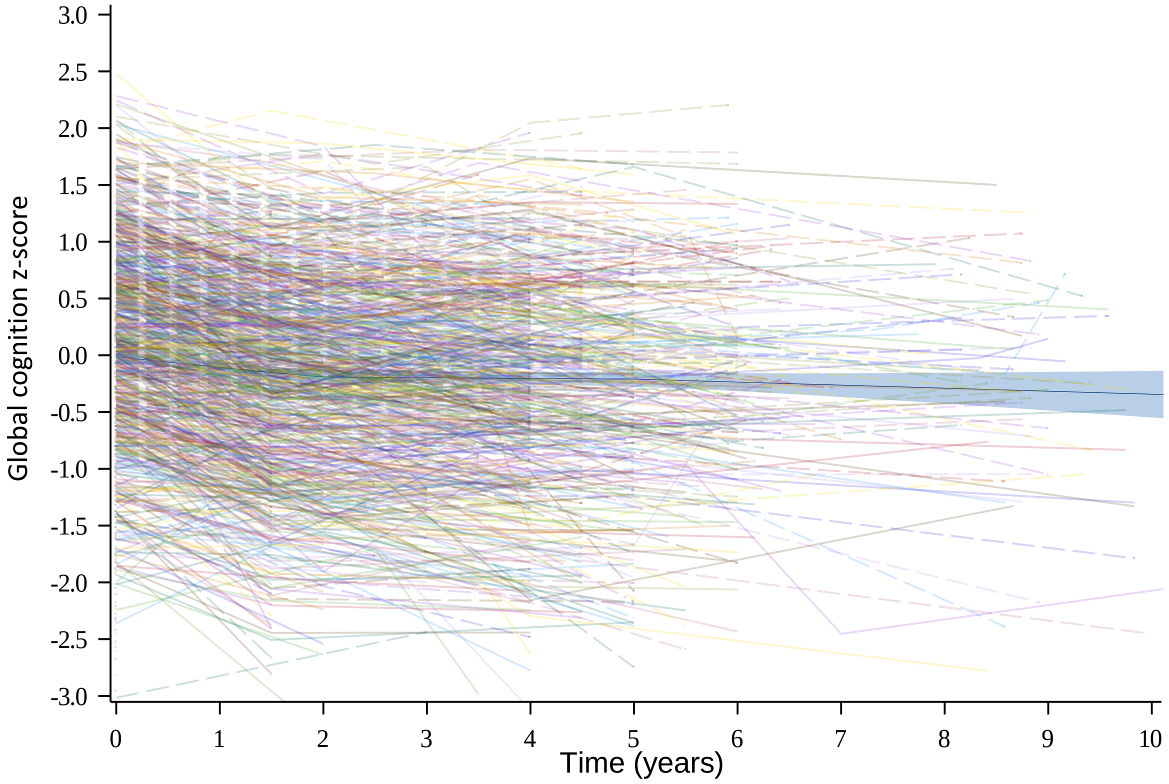

Estimated annual slope of z-score: -0.098, SE = 0.0037, p < 0.001; The blue band shows the 95% prediction confidence.
